# Testing the causality between *CYP9M10* and pyrethroid resistance using the TALEN and CRISPR/Cas9 technologies

**DOI:** 10.1038/srep24652

**Published:** 2016-04-20

**Authors:** Kentaro Itokawa, Osamu Komagata, Shinji Kasai, Kohei Ogawa, Takashi Tomita

**Affiliations:** 1 Department of Medical Entomology, National Institute of Infectious Diseases, 1-23-1 Toyama, Shinjuku-ku, Tokyo 162-8640, Japan; 2 Japan Agency for Medical Research and Development (AMED), 20F Yomiuri Shimbun Bldg. 1-7-1 Otemachi, Chiyoda-ku, Tokyo 100-0004 Japan

## Abstract

Recently-emerging genome editing technologies have enabled targeted gene knockout experiments even in non-model insect species. For studies on insecticide resistance, genome editing technologies offer some advantages over the conventional reverse genetic technique, RNA interference, for testing causal relationships between genes of detoxifying enzymes and resistance phenotypes. There were relatively abundant evidences indicating that the overexpression of a cytochrome P450 gene *CYP9M10* confers strong pyrethroid resistance in larvae of the southern house mosquito *Culex quinquefasciatus*. However, reverse genetic verification has not yet been obtained because of the technical difficulty of microinjection into larvae. Here, we tested two genome editing technologies, transcription activator-like effector nucleases (TALEN)s and clustered regularly interspaced short palindromic repeats (CRISPR/Cas9), to disrupt *CYP9M10* in a resistant strain of *C. quinquefasciatus*. Additionally, we developed a novel, effective approach to construct a TALE using the chemical cleavage of phosphorothioate inter-nucleotide linkages in the level 1 assembly. Both TALEN and CRISPR/Cas9 induced frame-shifting mutations in one or all copies of *CYP9M10* in a pyrethroid-resistant strain. A line fixed with a completely disrupted *CYP9M10* haplotype showed more than 100-fold reduction in pyrethroid resistance in the larval stage.

An important goal of insecticide resistance research is to identify the gene (or allele) responsible for the resistance phenotype. The identification mutation causing resistance permit the diagnosis of the resistance status of individual insects, even if dead. Although insecticide resistance can be conferred in several different ways, metabolic degradation of an insecticide by one or multiple detoxification enzymes and/or structural alteration of a target protein for the insecticide molecule are two major mechanisms. Identifying a gene responsible for resistance becomes more challenging in cases in which detoxifying enzymes confer the resistance because there are usually multiple genes potentially catalyzing detoxification of a given insecticide. Verification to narrow the range of candidate genes relies on accumulating more or less circumstantial evidence such as an enzyme’s catalytic ability, genetic linkage and association in natural population. An enzyme’s catalytic ability, by determining whether an enzyme coded by a candidate gene metabolizes a given insecticide, becomes a vital evidence but is often time-consuming to confirm, especially for genes encoding such as cytochrome P450s, for which there is no single reliable method to reconstitute a catalytically functional recombinant enzyme^1^. Thus, a failure to observe a catalytic activity *in vitro* usually does not convince a researcher to drop a gene from the candidate list. Furthermore, the presence of catalytic ability against an insecticide of interest cannot be the sole strong evidence. For example, the *CYP4H34* gene was overexpressed in a pyrethroid-resistant strain of the southern house mosquito *Culex quinquefasciatus*, and the recombinant protein also had catalytic activity to degrade pyrethroids^2^,^3^. However, neither the gene locus nor overexpression was genetically linked to the resistance phenotype^4^. Reverse-genetics to disrupt gene function could enhance such vilification for gene–phenotype causal relationships. RNA interference (RNAi) has been successfully used in some insecticide resistance studies^5^,^6^,^7^. The drawback of RNAi, however, is that it requires individual injections of double-strand RNA unless the species allows an alternative delivery pathway, such as oral administration. Microinjection generally causes severe damage to insects, which often do not tolerate the subsequent bioassay performed to detect change in insecticide susceptibility. Furthermore, the effect of RNAi is temporary, varying among species and tissues, and is more or less incomplete^8^.

Recent emerging technologies called genome editing offer a new way to disrupt gene function. Genome editing employs programmable artificial DNA endonucleases such as zinc-finger nuclease^9^, transcription activator-like effector nuclease (TALEN)^10^,^11^ and clustered regularly interspaced short palindromic repeats (CRISPR)/Cas9 system^12^, which are designed to target a specific sequence in a genome. These nucleases introduce a double-strand break at a targeted DNA site, which then undergoes a repair process called non-homologous end joining (NHEJ) within a nucleus. Because NHEJ is error-prone, it often leaves an indel mutation at the breakage site, thereby causing a frameshift mutation if an open reading frame is targeted. The important difference of genome editing from RNAi is that it directly mutates a gene in a genome. The function of the gene can be completely disrupted, and the mutation can be transmitted to offspring via a germline-mutation. We thereby create a population of insects homogeneous for a completely knocked-out allele of a detoxification enzyme gene of interest while the genetic background remained mostly unchanged from that of the original resistant strain. The population can be used to observe susceptibility to insecticide in scale such as a half-lethal dosage which usually requires treating hundreds of individuals to measure.

Currently, TALEN and CRISPR/Cas9 are most often used for genome editing experiments because of their design simplicity. A TALEN consists of a TALE domain, which binds a specific targeted DNA sequence, and a *Fok*I nuclease domain, which is responsible for nuclease function. Normally, two TALENs are required to induce a double-strand break in DNA as two *Fok*I domains are needed to form a homodimer. The specificity of the TALE domain to a DNA sequence is mediated by the tandem repeats of a small polypeptide (34 amino acid residues). Each repeat shares the same amino acid sequence except for a doublet called repeat-variable di-residues (RVDs). Each repeat with different RVDs recognizes a different base on a nucleotide^13^,^14^. CRISPR/Cas9 has been introduced shortly after TALENs. CRISPR/Cas9 exploits the prokaryotic immune system’s machinery for destroying foreign DNAs such as bacteriophages or toxic plasmids. In the engineered CRISPR/Cas9 system, a single molecule RNA called guide RNA (gRNA) mediates the sequence recognition of DNA, and Cas9, an RNA-guided DNA endonuclease, cleaves the DNA sequence recognized by the gRNA by simple DNA–RNA base paring^12^.

In this study, we disrupted a detoxification enzyme gene in a pest insect using TALENs and CRISPR to validate the causality of an insecticide resistance phenotype. We also developed a novel method to construct TALEs using phosphorothioate chemistry (PC)^15^ (Fig. 1). As a proof of concept, we targeted *CYP9M10* in *C. quinquefasciatus. CYP9M10* encodes a cytochrome P450 that degrades pyrethroid insecticides *in vitro*^2^. *CYP9M10* is overexpressed in a strongly pyrethroid-resistant strain, JPal-per (JPP)^3^, and is linked to the resistance phenotype^18^. The upregulation of *CYP9M10* in the JPP strain is due to multiple *cis*-acting mutations and recent gene duplication^16^,^17^,^18^. Althouhg the two duplicated copies have the same sequence in the transcribed region, a junction formed by the tandem duplication of a 100-kb segment is located 1.1 kb upstream of the transcription start site of *CYP9M10* and differentiates the two copies (v1 and v2)^16^. Although, much evidences support the causal relationship between *CYP9M10* and pyrethroid resistance^2^,^16^,^17^,^19^, RNAi verification had not been conducted because the gene overexpression and resistance are specific to the larval stage, which is highly vulnerable to microinjection^20^. Both TALENs and gRNA targeting *CYP9M10* introduced frame-shift mutations in the JPP strain. We obtained lines fixed with each haplotype mutated in either v1 or v2 copies of *CYP9M10*, or both. Dramatic reduction (110-fold) in permethrin (one of pyrethroid insecticides) resistance was observed in a line in which all *CYP9M10* copies were disrupted.

## Results and Discussion

### Construction of TALENs using phosphorothioate chemistry (PC)

**Figure 1:**
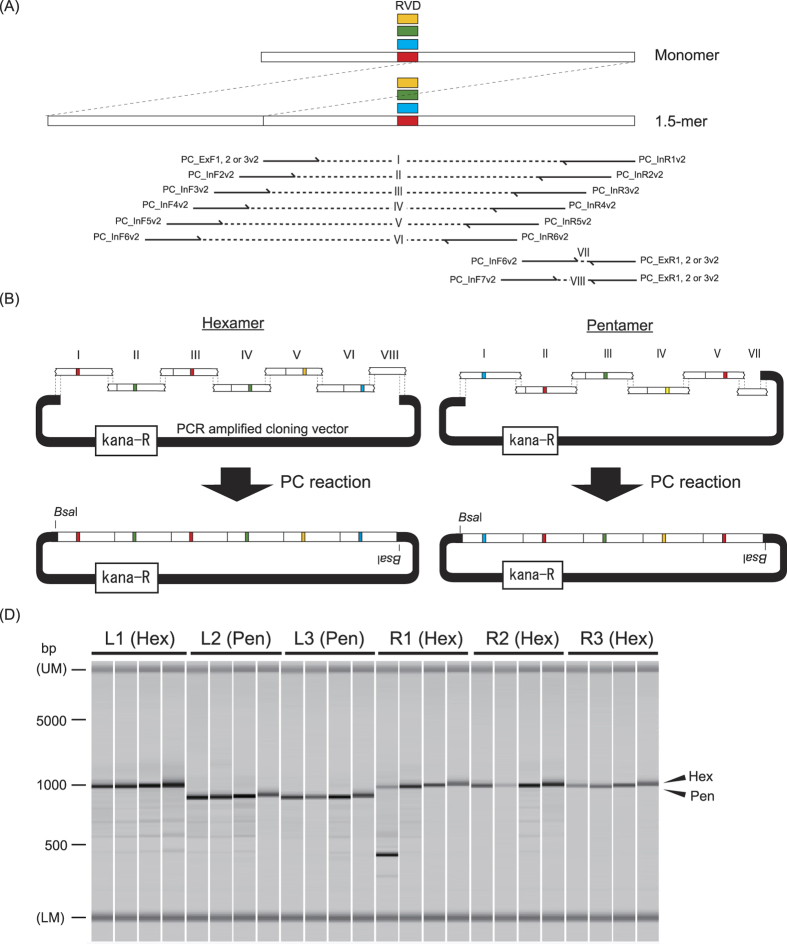
PC assembly of TALE hexamer/pentamer. (**A**) Structure of 1.5-mer template is shown. Phosphorothioate modified primers used were depicted blow. The sequences of the primers are listed in Table S1 in the supplemental online file. (**B**) PCR amplified monomers are assembled into hexamer or pentamer. The names of fragments (I–VIII) correspond to Fig. 1A. Note that the fragments VII and VIII work as adjusters for pentamer and hexamer assemblies, respectively. Each construct possesses *Bsa*I site at both ends which will be used for subsequent full length TALE construction by Golden Gate reaction. (**C**) Colony check PCR was conducted for clones of hexamer/pentamer for TALE9M10L & R assembled by PC. The PCR products were electrophoresed in a microchip electrophoresis system MCE-202 MultiNa (Shimadzu, Kyoto, Japan). “UM” and “LM” each indicate the upper and lower markers, respectively. Arrowheads on right side indicate expected fragment size for correctly assembled hexamers and pentamers.

Current widely-used, lab-scale TALE construction methods^21^,^22^ adopt hierarchical assembly approaches. At the first step (level 1 assembly), each five to 10 TALE monomers are ligated in precise order. At the second step (level 2 assembly), two to four such units are assembled into a backbone plasmid containing a functional enzyme domain (e.g. *Fok*I domain for TALEN), again in precise order, to construct a full length TALE. For ligating DNA fragments seamlessly in precise order, Golden Gate assembly^23^ is used. Golden Gate assembly involves a simultaneous reaction of a type IIS restriction enzymes that form desired overhang sequences at the ends, and ligase. Because the specificity of enzymatic ligation relying on a short overhang is relatively low, extensive optimization and checking of several colonies are often required. There are alternative construction approaches based on ligation-independent cloning (LIC) or uracil-specific excision reagent (USER)^24^,^25^,^26^ that use long single-strand DNA overhangs to avoid enzymatic ligation. Those techniques, however, require large numbers of initial plasmids and/or expensive commercial reagents. In the present study, we tested a novel technique for cloning a TALE hexamer or pentamer without an enzymatic reaction. Our method introduces two new ideas. One employs a cloning method based on PC^15^,^27^ in level 1 assembly. The PC reaction contains cleavage of phosphorothioester inter-nucleotide linkages mediated by iodine under alkaline conditions^28^ , creating an overhang long enough to mediate ligase-free cloning. A phosphorothioester linkage can be introduced directly into polymerase chain reaction (PCR) primers at relatively low cost by most oligonucleotide vendors. Moreover, the enzyme-free PC reaction is rapid and requires only inexpensive reagents. The second idea is the use of 1.5-mer plasmids as templates for monomer fragments (Fig. 1A). The 1.5-mer plasmids contain a full-length monomer plus the 3′ half of a monomer from the RVD. Because each 1.5-mer contains only one RVD, only four initial plasmids are required as PCR templates of the monomers. PCR primers are designed to amplify approximately 102 bp of a 1.5-mer while gradually shifting toward the 5′ direction by approximately 7 bp for every position in the hexamer (Fig. 1A). As a result, each monomer fragment automatically acquires unique overhang at both ends (Fig. 1B) with no additional sequence added to primers for inter-monomer connections. A small unit without RVD (VII or VIII in Fig. 1A,B) is added to fill the gap resulting from those shifts. This small unit also works as an ‘adjuster’, so that experimenter can construct pentamers (and possibly even smaller polymers) just by changing it (Fig. 1B). The amplified monomers and an amplified plasmid backbone were mixed and subjected to the cleavage reaction at 70 °C to create overhangs. The mixture was annealed just by dropping the temperature to RT in the same tube and then directly transformed into DH5a *E. coli* with no purification procedure. Three cloned hexamers are finally assembled into a backbone plasmid by conventional Golden Gate using *Bsa*I and ligase as level 2 assembly. The TALENs finally obtained by our methods were identical to those obtained by the TALE Toolbox method^21^ except for some synonymous codon changes. Our detailed protocol is described in a supplemental online file.

**Figure 2:**
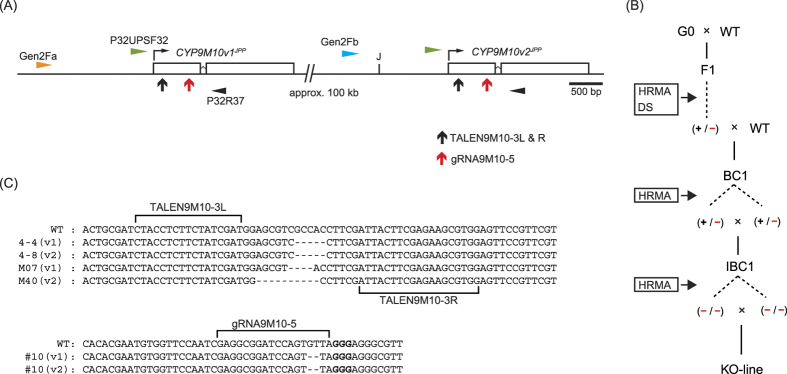
TALEN and CRISPR/Cas9 targets, fixation and fixed haplotypes. (**A**) Positions of TALEN9M10-3L & R and gRNA9M10-5 are shown as vertical arrowheads on translated region of *CYP9M10*. There are two *CYP9M10* copies separated by approximately 100-kb in the JPP strain. “J” indicates the junction formed by the tandem duplication. Primers used for the SNiPerase assay and direct sequencing analysis were shown as horizontal arrowheads. (**B**) Crossing scheme to fix a mutant haplotype is diagrammed. “WT” indicates wild-type JPP counterpart for mating. “BC1” is backcross 1, and “IBC1” is inbred BC1. The box indicates genotyping method used in each step, as “HRMA” indicates high resolution melting analysis and “DS” indicate direct sequencing. (**C**) Haplotypes finally fixed in each knockout (KO) line. Bold characters indicate PAM for gRNA9M10-5.

Four TALE hexamers and two pentamers for TALEN9M10-3L & R (Fig. 2A) were assembled and cloned. As sizes of inserts were checked by colony PCR for 24 clones (four clones of each hexamer/pentamer), all except one (96%) hexamer clone showed single fragments with expected sizes (0.9 and 1.0 kb, respectively) (Fig. 1C). Among the 23 clones with inserts of correct-size, 22 (96%) had correct sequences. One hexamer clone contained a point mutation, which probably occurred during PCR. The correctly assembled clones of each hexamer/pentamer were used to assemble TALENs by Golden-Gate reaction using *Bsa*I. The PCR amplified monomer fragments can be stored for at least six-months at −20 °C without an obvious decrease in cloning efficiency.

The high accuracy and robustness of PC will add a new option for TALE construction methods. The PC method normally takes 5 days to yield a TALEN plasmid, the same time as that needed for Golden Gate construction^22^, and 1 day longer than TALE Toolbox method^21^.

### Knocking-out *CYP9M10* with TALENs

TALENs, TALEN9M10-3L and -R, targeting the first exon of *CYP9M10* in JPP (Genbank ID: AB332027) were designed with the SAPTA algorithm^29^ and constructed using the PC assembly described above. Frame-shifting in this exon was expected to disrupt the gene function because critical protein domains such as a heme-binding region are located downstream. Because *CYP9M10* is duplicated in the JPP strain^16^, there were two potential targets of the TALENs (one each on v1 and v2) 100 kb apart (Fig. 2A).

We first injected plasmid DNA (400 ng/μl) harboring the *Aedes aegypti* polyubiquitin promoter^30^ upstream the TALEN construct into embryos of the JPP strain as this promoter has been successful in *A. aegypti*^31^. However, we observed only slight signs of mutations in G0s and no mutant offspring. We then synthesized messenger RNAs (mRNAs) of the TALENs *in vitro* and injected directly into approximately 100 embryos of the JPP strain. Some of the injected embryos that did not hatch (>50%) were used to check the efficiency of the TALENs that we designed. In a heteroduplex cleavage enzyme (SNiPerase) assay, 16 of 21 embryos (76%) showed expected heterodimer digestion patterns (Fig. S1).

Five of six G0 males that reached adulthood showed somatic mosaicisms in the SNiPerase assay. Those five males were crossed randomly with 20 virgin wild-type (WT) JPP females in the same cage. In the pooled F1 offspring from these females, males and females were separated in pupae. Ninety-six males were genotyped by high resolution melting analysis (HRMA) using a hind-leg, and heterozygous insects were further analysed by direct sequencing (Fig. 2B). Eleven males among them inherited a mutation (Table 1). Those individuals carried mutation at either of the two loci (v1 or v2) but no individuals carrying mutations at both loci were observed. Finally two haplotypes that were mutated in either the v1 or the v2 copy (M04 and M40, respectively, in Fig. 2C) were chosen for advancing to fixation (Fig. 2B).

Four G0 females out of five that reached to adulthood, on the other hand, showed visible mutation mosaicism. Two of the four females yielded at least one F1 offspring carrying a mutation detected by HRMA (Table 1). From one of the two broods, two haplotypes that were mutated in either the v1 or the v2 copy (4–4 and 4–8, respectively, in Fig. 2C) were chosen to advance for fixation. No individuals carrying mutations at both loci were observed from those broods.

### Knocking-out *CYP9M10* with CRIPR/Cas9

The protospacer sequence of the gRNA targeting *CYP9M10*, gRNA9M10-5, was determined by visual inspection for the presence of NGG protospacer adjacent motif (PAM) in the first exon of *CYP9M10* (Fig. 2A). The gRNA was synthesized and injected into approximately 100 embryos of the JPP strain along with commercially obtained hCas9 mRNA. Unhatched embryos were used to check for mutations by SNiPerase. Of the 24 unhatched embryos after the injection, 11 showed expected heteroduplex digestion patterns (46%) (Fig. S1).

One of the six G0 males that reached to adulthood showed somatic mosaicism. This single male was crossed with four WT JPP females. Pooled F1 offspring from these females included larvae inheriting mutations. In total, 128 F1 males were genotyped, and four males inherited germline mutation. Among them, one male had mutations at both the v1 and v2 loci (#10 in Fig. 2C and Table 1). This male was crossed with WT JPP females and advanced to fixation. Four G0 females that emerged were crossed with WT JPP males and allowed to oviposit without being checked for somatic mosaicism. No mutant F1 offspring from those females, however, was detected (Table 1).

### *CYP9M10* knockout (KO) lines

**Figure 3:**
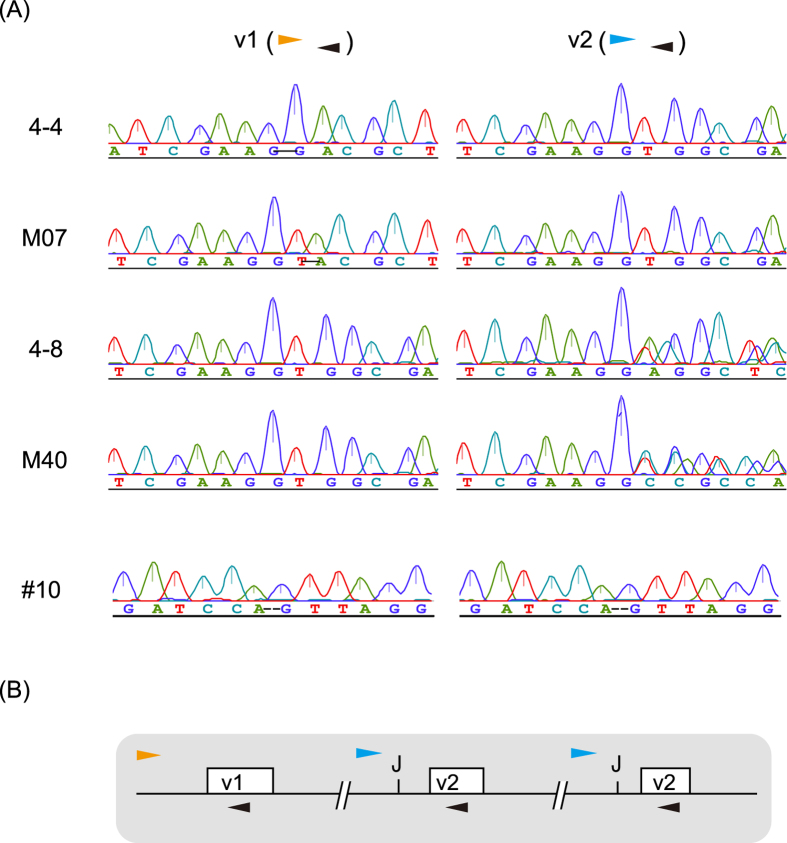
Sequence pherograms of each fixed KO line. (**A**) The v1 copy and v2 copies of *CYP9M10* were directly sequenced for each fixed KO line. The horizontal arrowheads indicate primers used to amplify each fragment as colours corresponding to Fig. 2A. (**B**) Showing a model for three *CYP9M10* copies (two v2 copies).

The genotype frequencies at the IBC1 generation in the 4–4 line during the fixation process (Fig. 2B) departed significantly from the expected ratio (1:2:1) of the normal Mendelian inheritance model (Table 2 and Fig. S2), suggesting that the knocked-out *CYP9M10* haplotype was linked to lower survival in this line. Since the other lines did not show such a large departure from the Mendelian model, it is not clear whether the lower survival in the 4–4 line was due to the knockout of *CYP9M10*. Several generations after establishment of KO lines, the v1 and v2 copies were separately amplified and sequenced directly for four individuals in each line. All four individuals in the M07 and 4–4 lines each carrying the disrupted v1 copy showed sequence pherograms with homozygous peaks for both v1 and v2 copies. In contrast, all four individuals in the M40 and 4–8 lines each carrying a disrupted v2 copy still showed heterozygous poly peaks composed of the mutated and WT alleles (Fig. 3A) in the v2 fragment. We checked another 16 individuals (eight females and eight males) from each M40 and 4–8 line after further several inbreeding generations and confirmed all individuals still showed same heterozygous sequence pherograms for v2 fragment as seen in Fig. 3A. Thus, the heterogeneity was not due to incomplete fixation (p < 0.0004 by the chi-square test for Hardy–Weinberg equilibrium) but rather indicating the presence of two v2 copies (thus, three copies of *CYP9M10* in total) per one haplotype, at least in those lines (Fig. 3A), with only one of the two having been mutated. Our previous estimate of the *CYP9M10* copy number in the JPP strain was two^16^,^19^. This discrepancy was probably simply due to the technical difficulty of distinguishing between two and three copies (a 1.5-fold difference) by quantitative PCR-based methods. Another, but non-mutually exclusive, hypothesis is that the copy numbers are polymorphic within the JPP strain because of instability of tandem duplication due to unequal crossing-over or replication slippage. Targeting two sites in the same chromosome concurrently may also induce a *de novo* duplication event by inter-chromosomal or -chromatid ligation^32^. Both the v1 and v2 fragments amplified from the four individuals in #10 line created by CRISPR/Cas9, on the other hand, showed homozygous peaks for the mutated alleles (Fig. 3B). At present we cannot determine the precise copy number status in the original JPP strain. Nevertheless, at least the M40 and 4–8 lines still carried two intact copies of *CYP9M10* and the #10 line was considered to no longer harbor a functional *CYP9M10* copy.

The TALEoffer program^33^ predicted one notable off-target site for TALEN9M10-3s in a *CYP9M10* pseudogene located upstream of the true *CYP9M10* locus (Fig. S3). No mutation, however, was detected within 200-bp range from the potential off-target site in any of the four KO-lines created by the TALENs.

### Effect on permethrin susceptibility

**Figure 4:**
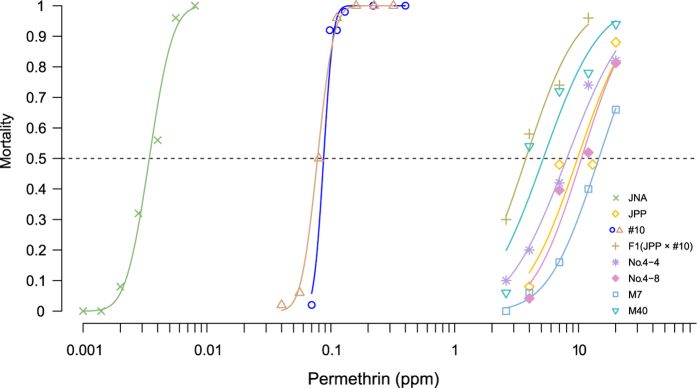
Permethrin susceptibility. The results of permethrin susceptibility assay are plotted. For #10, the assay was conducted at two different generations. The lines show the cumulative distribution function of a normal distribution function regressed on the result by the probit method.

Permethrin (one of pyrethroids insecticide) susceptibility was measured in fourth-instar larvae and compared to that of the parental JPP strain and pyrethroid-susceptible JNA strain (Fig. 4). The KO lines (M04, M07, 4–4 and 4–8) with one of the three (or two) copies disrupted showed little difference in susceptibility compared to the WT JPP strain. In contrast, #10, which carried no functional *CYP9M10* copy showed an approximately 110-fold reduction in permethrin susceptibility from that of the WT JPP. The susceptibility was not recovered completely to the susceptible level (the JNA strain) because the JPP strain also carries a *kdr* mutation (L1014F) in the voltage-gated sodium channel gene^34^,^35^, which is another genetic factor conferring pyrethroid resistance.

The absence of clear effect in M04, M07, 4–4 and 4–8 can be attributed to the reduction in gene dosage (1/2 in the two copies model, or 1/3 in the three copies model) that was too small to observe the phenotypic effect in such a toxicological bioassay. For instance, the F1 hybrid between #10 and JPP, which was considered to have a gene dosage exactly half that of the WT JPP, retained high resistance (only 2.7-fold reduction) (Fig. 4).

## Conclusion

Using genome editing technology, we obtained lines in which one or all copies of *CYP9M10* in a *C. quinquefasciatus* pyrethroid-resistant strain, JPP, were disrupted. Disruption of all the copies of *CYP9M10* in JPP reduced the pyrethroid resistance dramatically. This result constitutes strong evidence that *CYP9M10* is a major factor responsible for the strong pyrethroid resistance in JPP. As shown in our study, genome editing technologies allow rapid and direct verification of the causal relationship between a detoxifying gene and resistance phenotype even for species where RNAi experiments achieve limited success. The technology may be used for disrupting not only gene function but also promoters or regulatory elements suspected to be responsible for overexpression, if these sites can be targeted by any programmable nucleases.

In this study, we obtained only one haplotype (and line) in which all copies of amplified *CYP9M10* copies were disrupted only by CRISPR/Cas9 system. TALEN, on the other hand, produced more haplotypes disrupted only a single copies of *CYP9M10* than CRISPR/Cas9 (Table 1). It is not concluded, however, that either technology is simply more effective than the other for disruption of detoxification genes in our study because efficacies of both TALEN and CRISPR/Cas9 depend on target sequences, dosages, and perhaps by genes. Rather, an experimenter should choose a technology in consideration of construction cost and existence of potentially good target site. The result also showed an important drawback of genome editing that chance of simultaneous disruption of more than two loci is small compare to a single disruption event. Therefore, it would become more challenging to disrupt genes with extensive copy number change (more than 3-fold), which is often seen in detoxifying enzyme genes responsible for insecticide resistance^36^,^37^,^38^, enough to observe phenotypic change. Also, some insect pests, such as cockroaches, are not amenable embryonic microinjection. Despite of those drawbacks and some limitations, genome editing technologies will greatly enhance the process of verifications for involvement of detoxification enzyme in insecticide resistance, and help to extend our knowledge in this field.

## Methods

### Insects

The pyrethroid-resistant strain JPP was established from a population of *C. quinquefasciatus* collected from Saudi Arabia in the 1980s^39^. The strain was subjected to laboratory selection with permethrin for 20 consecutive generations^39^. The JNA strain is a pyrethroid-susceptible strain isolated from a strain called JHB^19^,^40^. Mosquitoes were kept in an insectarium on 25 °C. They were fed insect food (Oriental Yeast, Tokyo, Japan) during the larval stage and sugar water during the adult stage. Female mosquitoes were blood-fed on tethered mice overnight to initiate egg production. All experimental protocol using vertebrate animal in this study were reviewed and approved by the Animal Ethics Committee of NIID. The experiments were conducted in accordance with the approved guideline.

### Primers

All oligonucleotides used were described in Table S1 in the supplemental online file.

### Plasmid construction

The plasmid kit^21^ for TALEN construction was gifted by Feng Zhang (Addgene kit #1000000019). The gRNA cloning vector was constructed by modification of a gRNA_cloning vector^41^ gifted by George Church (Addgene plasmid #41824).

The 1.5-mer template plasmids were constructed from monomer template plasmids pNI_v2, pNG_v2, pNN_v2 and pHD_v2 as follows. A whole plasmid including the full length monomer was amplified with the primers TALE_monoBB_R and TALE_monoBB_F. A half monomer (3′ half from the RVD) was amplified by primer TALE_monoIn_F and TALE_monoIn_R from pNI_v2. Each whole plasmid fragment and a half monomer fragment was ligated by *Bsa*I and ligase after *Dpn*I (Takara, Oosaka, Japan) treatment, and then cloned in DH5α *E. coli*. The AaPUb-pTAELN_v2 plasmids were constructed from the pTAELN_v2 plasmids by replacement of the original CMV promoter with the *Aedes aegypti* polyubiquitin (AaPUb) gene promoter^30^. The AaPUb gene promoter was amplified from genomic DNA of the *A. aegypti* Liverpool strain with primers AAEpUb_BsaI-CTAG_F and AAEpUb_SacI_R. The fragment was digested with *Bsa*I and *Sac*I and then cloned between the *Spe*I and *Sac*I sites of the pTAELN_v2 plasmids.

The backbone plus the structural part of gRNA of the gRNA_cloning vector was amplified with the primers gRNA_CRISPR_F and _R. *Drosophila* U6 (DmU6) promoter^42^ was amplified with primers U6-Fout and -Rout from genomic DNA of the Oregon-R strain. The *LacZ* gene was amplified from pUC19 plasmid with primers LazZ-CRISPR_F and LazZ-CRISPR_R. The DmU6-gRNA cloning vector was constructed by ligating those three fragments using *Bsa*I and ligase, which was cloned in DH5α *E. coli*. Since the microinjection of DmU6 promoter plasmid, however, did not work efficiently in our experiment (data not shown), the T7-gRNA cloning vector was finally constructed by self-ligating the fragment amplified with primers PCR_R2 and T7-BsaI-LacZ from the DmU6-gRNA cloning vector.

### Assembly of TALENs and mRNA synthesis

Oligonucleotides with phosphorothioate internucleotide linkages were synthesized by Eurofins Genomics Co., Ltd. (Tokyo, Japan). The detailed protocol for PC assembly is described in separate online material. Constructed hexamer and pentamer plasmids were assembled into the backbone plasmid pTALEN_v2^21^ by Golden Gate reaction for full length TALENs. For mRNA synthesis, each TALEN plasmid was cut with *Stu*I and subjected to mMESSAGE mMACHINE T7 ULTRA Transcription Kit (Thermo-Fisher Scientific, MA, US). The LiCl precipitation solution contained in the kit was used to purify the mRNA.

### Guide-RNA construction

Oligonucleotides T7gRNA9M10-5 (+) and T7gRNA9M10-5 (−) were annealed and cloned between the *Bsa*I site of the T7-gRNA cloning vector. For *in vitro* RNA synthesis, the plasmid cloned with gRNA9M10-5 was digested by *Xba*I and subjected to RNA synthesis by a T7 High Yield RNA Synthesis Kit (NEB, MA, US). The transcribed gRNA was purified with NucleoSpin miRNA Plasma Kit (Macherey-Nagel, NRW, Germany).

### Microinjection

The microinjection method was similar to other mosquito egg microinjection methods^43^,^44^ with some modifications. The injection needles were prepared by pulling aluminosilicate glass capillaries with filament (1.0 mm internal diameter) (Sutter, CA, US) using a micropipette puller P-1000 (Sutter). Microinjection solution for TALENs contained 330 ng/μl of each TALEN mRNA. The solution for CRISPR/Cas9 contained 150 ng/μl gRNA described above and 200 ng/μl of PrecisionX Cas9 SmartNuclease mRNA (System Bioscience, CA, US). To the microinjection solution was added 0.1 volume of 0.05% Brilliant Blue FCF (Wako, Oosaka, Japan) solution followed by filtering through a 0.22-μm spin filter (Millipore) before use. Microinjection was performed in embryos 1–2-h post-oviposition. The embryos were aligned horizontally with posterior ends facing to the same direction and then attached to double-sided sticky tape attached on a cover glass by the other side. The embryos were covered with 20% carboxylmethyl cellulose (Wako) solution containing 25% ethanol during the injection, which was performed to the posterior end of each embryo under microscope. Air pressure was supplied from an N_2_ bottle and controlled with a microinjector IM-300 (Narishige, Tokyo, Japan). The injected embryos were washed gently with distilled water and submerged in water kept at 25 °C until hatching.

### SNiPerase assay

Genome DNA were extracted from insects by alkaline lysis^45^. From embryos, each single egg was placed on the bottom of a 0.2-ml microtube and crushed in 5 μl of 0.2-M NaOH solution using a disposable micropipette tip. From living adults, on the other hand, a hind leg of each mosquito was placed in 0.2 ml microtube and homogenized in 10 μl of 0.2-M NaOH using a bead shaker. The homogenates were incubated at 75 °C for 10 min, neutralized by adding an equal volume of 360-mM Tris-HCl (pH 8.0) and diluted 10 times with H_2_O. PCRs were performed with ExTaq polymerase (Takara, Shiga, Japan) in 45 cycles using primers P32UPSF32 and P32R37 (Fig. 2A). PCR products were subjected to the SNiPerase (Frontier Genomics, AK, USA) reaction as described in manufacturer’s protocol. The reaction was stopped by addition of 10× loading dye (Takara) containing 0.9% SDS and the products were electrophoresed in 2% agarose gel. The DNA in the gel was visualized with ethidium bromide and pictured under UV light.

### High resolution melting analysis (HRMA)

HRMA^46^ was used for genotyping individuals. The gDNA prepared by the above method was used as a template. The reaction mixture constituted of 10-μl KOD-FX PCR mixture (TOYOBO, Osaka, Japan) supplemented with 0.5-ul of 20× EvaGreen dye (Biotium, CA, USA). The primer pairs used for TALEN9M10-3 and gRNA9M10-5 targeted sites were TALEN_9M10-3FA_F × TALEN_9M10-3FA_R and CRISPR_9M10-5_FA_F2 × CRISPR_9M10-5_FA_R2, respectively. PCR was performed in a PikoReal real-time PCR machine (Thermo-Fisher Scientific) at 95 °C for 2 min followed by 40 cycles of 98 °C for 5 s and 60 °C for 60 s. A melting curve was then recorded from 60 to 95 °C with 0.2 °C increment intervals. Data was analysed with PikoReal Software 2.2 (Thermo-Fisher Scientific).

### Direct sequencing

The two copies of *CYP9M10, -v1* and -*v2* (Genbank IDs: AB551111 and AB551112, respectively), were separately amplified by KOD-FX polymerase using primers Gen2Fa−P32R37 and Gen2Fb−P32R37, respectively (Fig. 2A). Direct sequencing was performed using the primer P32F44 (Fig. 2A) for both fragments in ABI3130 capillary sequencer (Applied Bioscience). The heterozygous sequencing pherogram was analysed with Poly Peak Parser^47^ (http://yosttools.genetics.utah.edu/PolyPeakParser/) to resolve a sequence of mutated allele.

### Identifying and fixing knockout mutant haplotypes

Each mutated haplotype was fixed in isofemale or isomale line (Fig. 2B). G0 females were crossed with wild-type JPP males in the same cage. After mating and blood-feeding, females were individually isolated and allowed to oviposit in 50 ml flask containing water. The G0 females that oviposited eggs were subjected to SNiPerase assay to confirm mutation mosaicism. G0 males, in contrast, were checked for mutation by SNiPerase assay before mating and only individuals with obvious mutation mosaicism were mated with WT JPP females. Larvae in the F1 generation were checked for inheritance of germline mutation by HRMA. F1 insects in which a mutation was detected were further analyzed by direct sequencing as described above. F1 females were crossed with WT JPP males in the same cage and fed blood. The F1 males, on the other hand, were genotyped before mating. Only males heterozygous for mutation were individually isolated and each was mated with three to five WT JPP females. The BC1 progeny obtained from F1 females or males with mutation were further grown to adults. At this stage, both females and males were genotyped before mating and only heterozygous mutants were mated for inbreeding. In the next generation (inbred BC1 or IBC1), only adults homozygous for mutation were crossed. The resulting strains were fixed with mutant haplotypes each originating from a unique mutant haplotype in the G0 generation.

### Off-target prediction and sequencing

Potential off-target sites of TALEN9M10-3L and R in the *C. quinquefasciatus* genome^40^ were searched by TALENoffer program^33^. The options used were #N-Terminal first: false, #N-Terminal second: false, #Hetero-dimers only: false, #Architecture: min = 10, max = 30, #Filter: q = 0.4, #RVD specificities: NA. The detected off target site (Fig. S3) was amplified with the primers P32UPSF29 and P32flaR2 and sequenced from both ends.

### Permethrin susceptibility assay

Permethrin susceptibilities of KO-lines and WT strains were assayed in fourth-instar larvae of each mosquito population as described in Itokawa, *et al.*^16^. Briefly, 25 fourth-instar larvae were placed in a plastic cup containing 50 ml distilled water. Dilution series of insecticide solution were prepared by dissolving permethrin (91% purity, obtained from Sumitomo-Chemical, Japan) in ethanol. Fifty to 200 μl of a permethrin solution was added to the cup containing larvae to achieve each final concentration. Ideal concentration ranges were determined by preliminary experiments using broad concentration interval for the KO-lines, and previous experiment^19^ for the WT strains. Two cups (i.e. 50 larvae) were used for each concentration of each group. Control cups were added 200 μl ethanol. Mortalities were measured by counting dead or paralyzed larvae after kept on RT for 24 h. Because we observed little mortality in the control of all groups (≤2%), the Abbott’s correction was not conducted. The result of the bioassay were regressed to the probit model by *glm *() function in the statistical software R version 3 (R Development Core Team, https://www.r-project.org/).

### Statistical analysis

Statistical analyses were performed with R version 3.

## Additional Information

**How to cite this article**: Itokawa, K. *et al.* Testing the causality between *CYP9M10* and pyrethroid resistance using the TALEN and CRISPR/Cas9 technologies. *Sci. Rep.* **6**, 24652; doi: 10.1038/srep24652 (2016).

## Supplementary Material

Supplementary Information

## Figures and Tables

**Table 1:** Inheritance of mutation derived by TALENs and CRISPR/Cas9.

TALENs	G0 adult	♂ × 5	♀	♀		
	F1 mutant	11/96♂	1/24♀	10/24♀		
	Derived KO line	M07, M40		4–4, 4–8		
**CRISPR/Cas9**	**G0 adult**	**♂**	**♀**	**♀**	**♀**	**♀**
	F1 mutant	4/128♂	0/24♀	0/24♀	0/24♀	0/24♀
	Derived KO line	#10				

**Table 2:** Genotype frequencies in IBC1 generation.

Genotype	M40			M07			4–4			4–8			#10		
	♀	♂	Total	♀	♂	Total	♀	♂	Total	♀	♂	Total	♀	♂	Total
+/+	12	15	27	11	13	24	14	17	31	7	9	16	9	10	19
+/−	22	21	43	28	28	56	28	25	53	25	28	53	27	27	54
−/−	14	12	26	9	7	16	6	6	12	16	11	27	12	11	23
*Χ*^2^*			1.06			4.0			8.6			3.6			1.8
*p*			0.59			0.14			0.014			0.17			0.40

*H_*0*_: The ratio of +/+, +/− and −/− is 1:2:1.
