# Supplementary material for: Testing the causality between CYP9M10 and pyrethroid resistance using the TALEN and CRISPR/Cas9 technologies
Source: Sci Rep. 2016 Apr 20;6:24652. doi: 10.1038/srep24652 (PMC4837413; doi:10.1038/srep24652)
Supplement: Supplementary Information [file srep24652-s1.pdf]

Supporting materials

Testing the causality between *CYP9M10* and pyrethroid resistance using the TALEN and CRISPR/Cas9 technologies

Kentaro Itokawa[1, 2], Osamu Komagata[1], Shinji Kasai[1], Kohei Ogawa[1] and Takashi Tomita[1]*

1. Department of Medical Entomology, National Institute of Infectious Diseases, 1-23-1 Toyama, Shinjuku-ku, Tokyo 162-8640, Japan
2. Japan Agency for Medical Research and Development (AMED), 20F Yomiuri Shimbun Bldg. 1-7-1 Otemachi, Chiyoda-ku, Tokyo 100-0004 Japan

*Corresponding Author: Takashi Tomita, Department of Medical Entomology National Institute of Infectious Diseases, Toyama 1-23-1, Shinjuku-ku, Tokyo 162-8640, Japan

Tel: (81)-3-52851111; Fax: (81)-3-52851147; E-mail: tomita@nih.go.jp

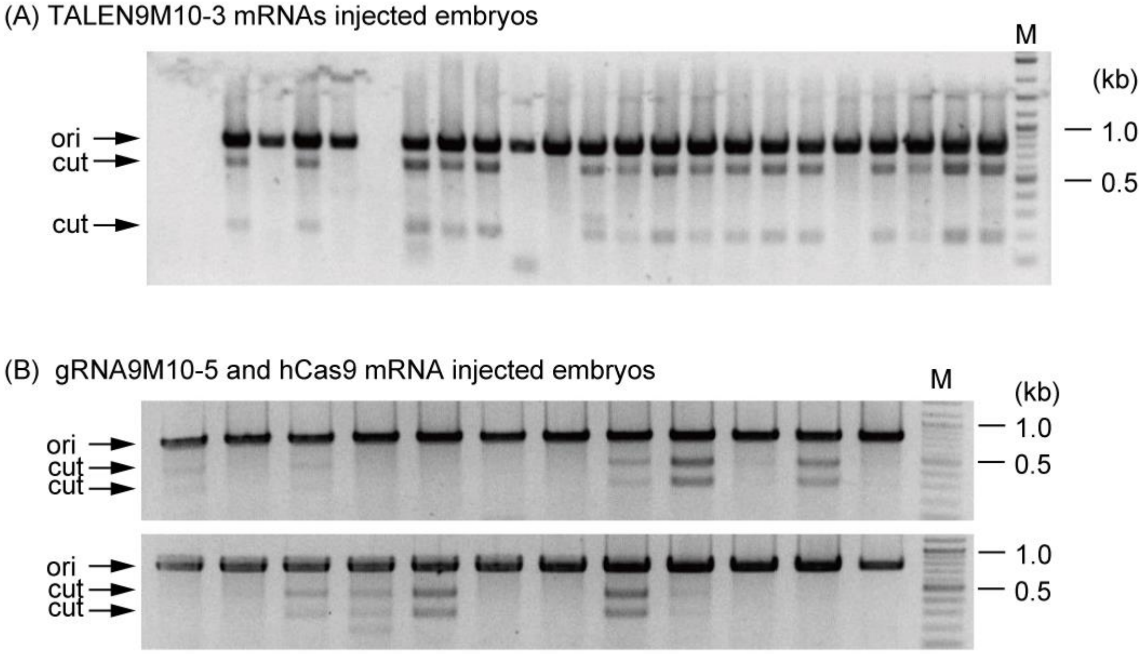

Fig. S1 Results of SNiPerase assay

SNiPerase assay was performed with unhatched embryos approximately 48-h post-oviposition. 'ori' indicates the size of the uncut PCR fragment. 'cut' indicates expected sizes of fragments generated by heteroduplex cleavage.

IBC1 M40

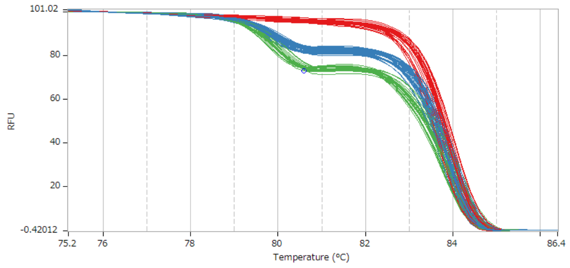

IBC1 M07

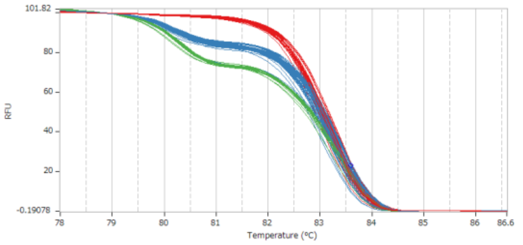

IBC1 4-4

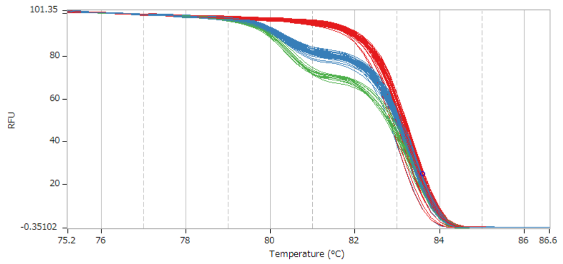

IBC1 4-8

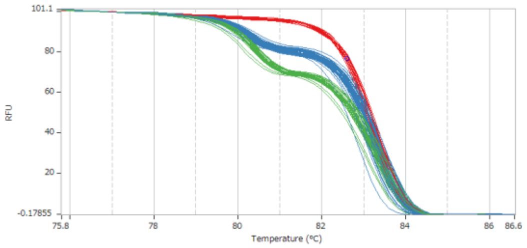

IBC1 #10

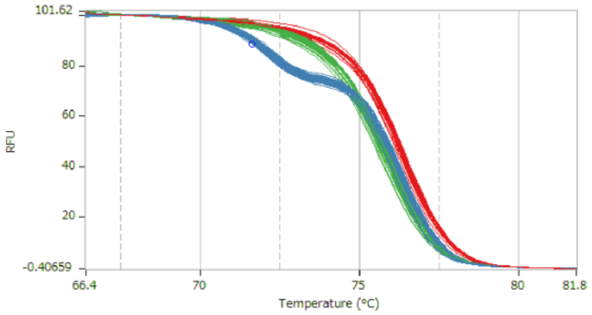

Fig. S2 Melting curves in HRMA genotyping

Melting curves during genotyping of the IBC1 population of each line. Red, blue and green indicate homozygotes of the WT haplotype, heterozygotes, and homozygotes of a mutant haplotype, respectively.

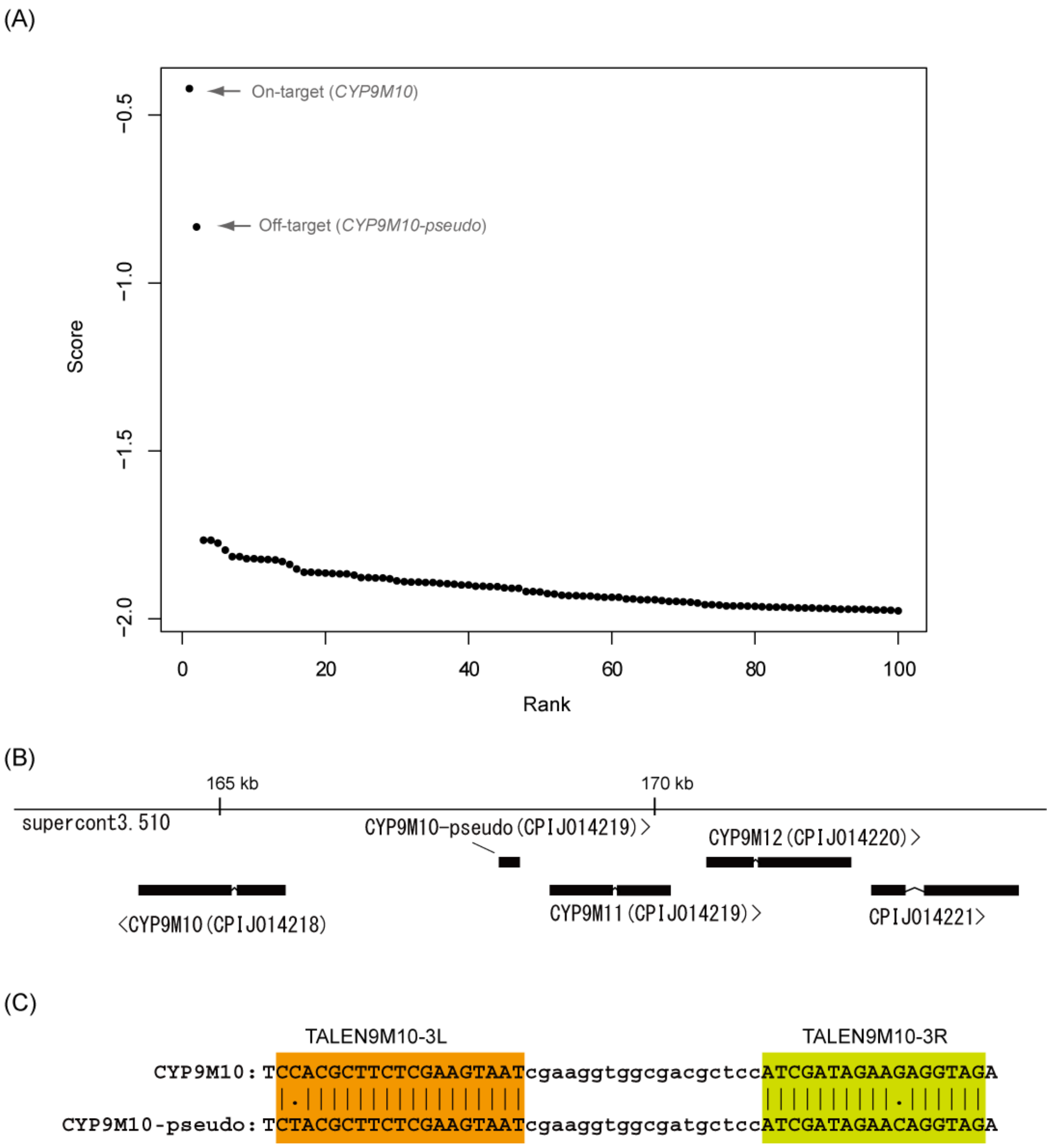

Fig. S3 Potential off-target site for TALEN9M10-3

(A) Top 100 potential off-target sites in the C. quinquefasciatus genome (Arensburger et al, 2010) scored by TALENoffer (Grau et al, 2013). The options used were #N-Terminal first: false, #N-Terminal second: false, #Hetero-dimers only: false, #Architecture: min = 10, max = 30, #Filter: q = 0.4, #RVD specificities: NA. (B) The position of *CYP9M10-pseudo* in the scaffold supercont3.510. (C) Sequence comparison between *CYP9M10* on-target site and *CYP9M10-pseudo* potential off-target site.

References

Arensburger P, Megy K, Waterhouse RM, Abrudan J, Amedeo P, Antelo B *et al* (2010). Sequencing of *Culex quinquefasciatus* establishes a platform for mosquito comparative genomics. *Science* **330**: 86-88.

Grau J, Boch J, Posch S (2013). TALENoffer: genome-wide TALEN off-target prediction. *Bioinformatics* **29**: 2931-2932.

Table S1

Name	Sequence	Note
PC_InF2v2	AG*CG*CA*CG*G*A*CTCACCCCA	For amplyfying monomer
PC_InF3v2	TG*TG*CC*AA*G*C*GCACGGACT	For amplyfying monomer
PC_InF4v2	CC*TG*TG*CT*G*T*GCCAAGCGCAC	For amplyfying monomer
PC_InF5v2	GT*TG*CT*GC*C*T*GTGCTGTG	For amplyfying monomer
PC_InF6v2	TC*CA*GA*GG*T*T*GCTGCCTGT	For amplyfying monomer
PC_InF7v2	GA*AA*CC*GT*C*C*AGAGGTTGC	For amplyfying monomer
PC_InR1v2	TC*CG*TG*CG*C*T*TGGCACAG	For amplyfying monomer
PC_InR2v2	GC*TT*GG*CA*C*A*GCACAGGCA	For amplyfying monomer
PC_InR3v2	AC*AG*CA*CA*G*G*CAGCAACCT	For amplyfying monomer
PC_InR4v2	AG*GC*AG*CA*A*C*CTCTGGAC	For amplyfying monomer
PC_InR5v2	AA*CC*TC*TG*G*A*CGGTTTCGAGT	For amplyfying monomer
PC_InR6v2	GG*AC*GG*TT*T*C*GAGTGCCTG	For amplyfying monomer
PC_ExF1v2	CTG*AG*GT*CT*CA*C*T*GACCCCAGAGCAGGTCGTG	For amplyfying monomer
PC_ExF2v2	CT*GA*GG*TC*T*C*CTCACCCCAGAGCAGGT	For amplyfying monomer
PC_ExF3v2	CT*GA*GG*TC*T*C*TGACTCCAGAGCAGGTCGTGGCA	For amplyfying monomer
PC_ExR1v2	TGA*CG*GT*CT*CG*G*T*GAGTCCGTGCGCTTGGCACA	For amplyfying monomer
PC_ExR2v2	TG*AC*GG*TC*T*C*GAGTCAGTCCGTGCGCTTGGCACA	For amplyfying monomer
PC_ExR3v2	TG*AC*GG*TC*T*C*TGAGTCCGTGCGCTTG	For amplyfying monomer
PC_VectorF	GAG*AC*CG*TC*AA*A*A*GGGCGACACAA	For amplifying hexamer/pentamer cloning vector
PC_VectorRv2	GA*GA*CC*TC*A*G*CGAAGGGCGACAC	For amplifying hexamer/pentamer cloning vector
TALE_monoF	CATGCCGAACTCAGAAGTGA	For sequencing a cloned hexamer/pentamer
TALE_monoR4	CTGATTTTCCCTTTATTATTTTCGAG	For sequencing a cloned hexamer/pentamer
TALE-Seq-F1	CCAGTTGCTGAAGATCGCGAAGC	For sequencing 1-6 (Sanjana et al., 2012)
TALE-Seq-R3	CGACCTGCTCTGGAGTCA	For sequencing 7-12
TALE-Seq-R4	GAAAGCTGGGCCACGATTGA	For sequencing 13-18
TALE_monoBB_R	TTTGGTCTCTTTCAGCGAAGGGCGAC	For plasmid construction
TALE_monoBB_F	TTTGGTCTCTCGGACTCACCCCAGAGCAGG	For plasmid construction
TALE_monoIn_F	TTTGGTCTCTTGAAGGGGGAAAGCAGGCAC	For plasmid construction
TALE_monoIn_R	TTTGGTCTCTTCCGTGCGCTTGGCAC	For plasmid construction
AAEpUb_BsaI-CTAG_F	GTTGGTCTCTCTAGATCTTTACATGTAGCTTGTGCATTG	For plasmid construction
AAEpUb_SacI_R	TTTGAGCTCGTTGAAATCTCTGTTGAGCAGAAA	For plasmid construction
U6Fout	AACTCAGGCAGACGGAACTG	For plasmid construction
U6Rout	AGGAGCAATCACAGGTGAGC	For plasmid construction
LacZ-CRISPR_F	TTTCTTCTGAGACCCAGCTTGTCTGTAAGCG	For plasmid construction
LacZ-CRISPR_R	TTTAAACAGAGACCGCTGGCACGACAGGTTTC	For plasmid construction
gRNA-CRISPR_F	TTTGGTCTCTGTTTTAGAGCTAGAAATAGCAAGTTAAAAT AAGGCTAG	For plasmid construction
gRNA-CRISPR_R	GCCGGTCTCCGTTATCGGGGAAG	For plasmid construction
T7-BsaI-LacZ	TAATACGACTCACTATAGGTGAGACCCAGCTTGTC	For plasmid construction
pCR_R2	CACCGCGAAAATGACATC	For plasmid construction
T7gRNA9M10-5(+)	TAGGCGAGGCGGATCCAGTGTTA	For gRNA9M10-5 plasmid consnrtuction
T7gRNA9M10-5(-)	AAACTAACACTGGATCCGCCTCG	For gRNA9M10-5 plasmid consnrtuction
CRISPR_9M10-5_FA_F2	GTTCCAATCGAGGCGGAT	For HRMA on the gRNA9M10-5 targeted site
CRISPR_9M10-5_FA_R2	AAAAACAACGCCCTCCCT	For HRMA on the gRNA9M10-5 targeted site
TALEN_9M10-3FA_F	CTCGAGTGGTTGCTGCTGTT	For HRMA on the TALEN9M10-3 targeted site
TALEN_9M10-3FA_R	GAACCGGTTTCACGAACGGA	For HRMA on the TALEN9M10-3 targeted site
Gen2Fa	GATTGGGCAAACTTAGAAGACC	For amplifying *CYP9M10v1*
Gen2Fb	CGGGTGAGTGTGTAATAGGGTA	For amplifying *CYP9M10v2*
P32R37	CCACACCAAACGAGAGAGAAGT	For amplifying *CYP9M10v1* and *v2:* For SNiPerase assay
P32UPSF32	CACCTACATATTTAAGAACGCCG	For SNiPerase assay
P32F44	CGTGCTGGCAAAATTCGT	For direct sequencing
P32UPSF29	TGATGTACCGATGATGAATGAC	For amplifying *CYP9M10*-pseudo
P32flaR2	AAGGAACTAGTGCTTAGCCTGC	For amplifying *CYP9M10*-pseudo

* Asterisks indicate phophorothioate inter-nucletide linkages

PC TALE assembly protocol

Described below is the TALEN construction protocol we used in the present study. Most of the components used in our method were developed by Sanjana *et al* (2012). We highly recommend consulting their protocol as well.

Structure of 1.5-mer

Here is the sequence of the 1.5-mer.

GGGGGAAAGCAGGCACTCGAAACCGTCCAGAGGTTGCT

GCCTGTGCTGTGCCAAGCGCACGGACTCACCCCAGAGC

AGGTCGTGGCAATTGCGAGC******GGGGGAAAGCAGGC

ACTCGAAACCGTCCAGAGGTTGCTGCCTGTGCTGTGCCA

AGCGCACGGA

The marked line shows the 0.5-mer part added to the original monomer. Asterisks indicate nucleotides corresponding to the two codons encoding an RVD.

Preparation of monomers

Set up primers and template as described in Fig. P1. Prepare the following PCR mixture.

KOD -plus- ver2 (Toyobo)	0.6 μl (0.6 U)
10x PCR Buffer	3 μl
2-mM dNTPs	3 μl
25-mM $MgSO_4$	1.8 μl
5-μM primer	1.8 μl
1.5-mer plasmid	1 ng
H_2O	up to 30 μl

Conduct PCR with thermal program as follows.

94°C for 2 min

(30 cycles of)

89°C for 10 s

58°C for 15 s

68°C for 10 s

68°C for 1 min

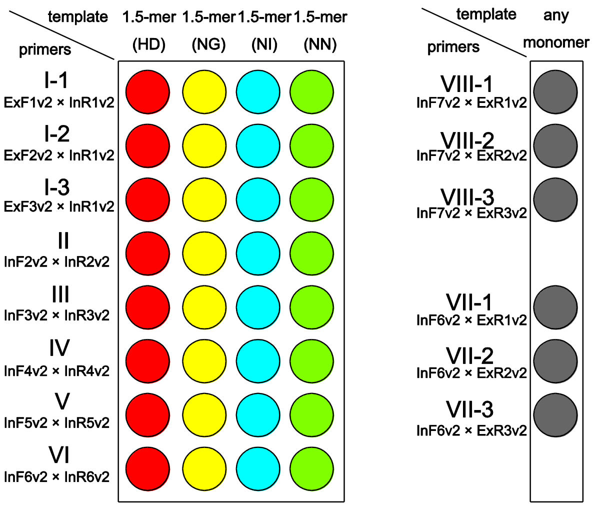

Fig. P1 Monomer panel setup

Electrophorese 1−2 μl of each PCR product to check yield and specificity.

During the electrophoresis, add 5 μl 1x PCR buffer containing 1 U of *Dpn*I (Takara) to each well. Then incubate as follows.

37 ºC for 1 h → 80 ºC for 20 min

Dilute each PCR product to approx. 7 ng/μl with tris-HCl (pH 8.0) containing 100-mM EDTA (65 μl in our case). The panel can be stored at −20°C.

Amplifying vector

Prepare the following PCR mixture.

KOD -plus- ver2	0.6 μl (0.6 U)
10x PCR Buffer	3 μl
2-mM dNTPs	3 μl
25-mM $MgSO_4$	1.8 μl
5-μM primer	
PC_VectorF	1.8 μl
PC_VectorRv2	1.8 μl

pNI_v2 (pJ201)	1 ng
H_2O	up to 30 µl

Conduct PCR with thermal program as follows

94°C for 2 min

(30 cycles of)

96°C for 10 s

60°C for 15 s

68°C for 2.5 min

68°C for 5 min

Add 1 U of *Dpn*I (Takara) and incubate as follows.

37°C for 1 h → 80°C for 20 min

Purify the PCR product on a column and dilute to 7 nM (approximately 12 ng/µl) with TE (pH 8.0). Store at –20°C.

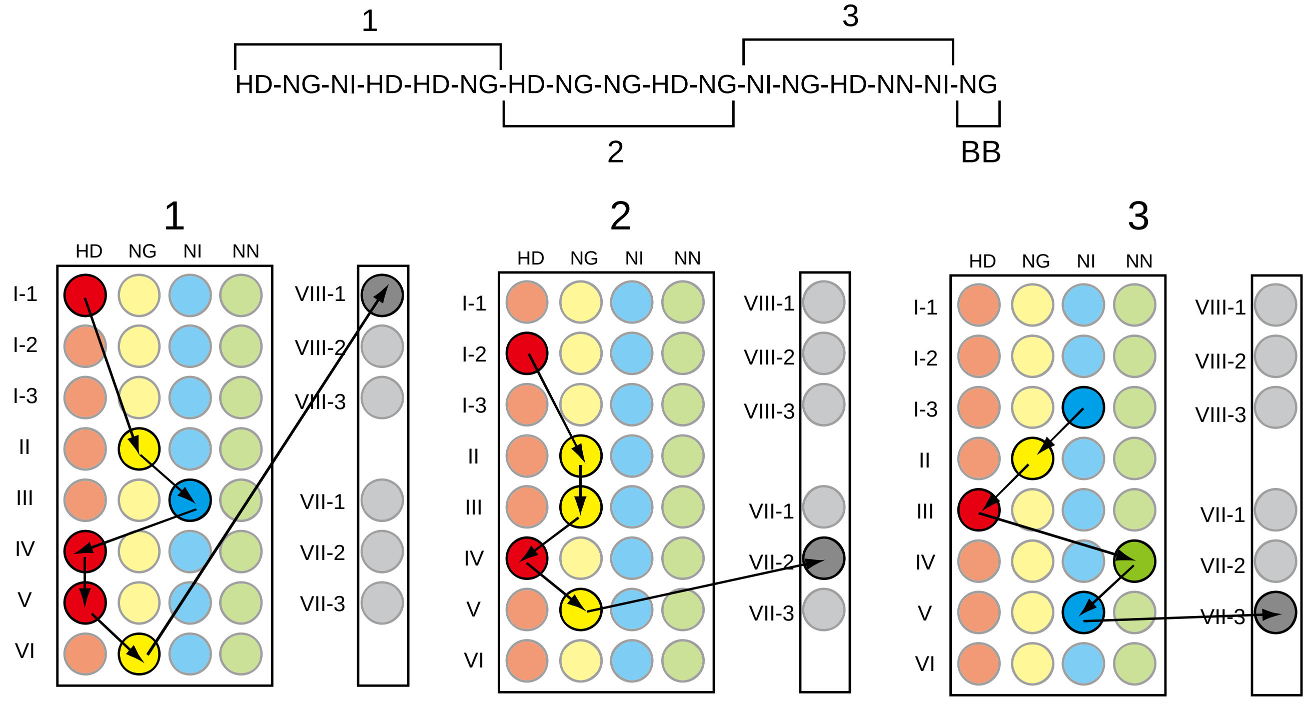

Fig. P2 Example for pipetting of monomers to construct one hexamer and two pentamers. Note that the last repeat will be included in the backbone (BB) vector (Sanjana et al., 2012).

Assembling hexamer/pentamer

Mix the solutions below. Fig. P2 shows an example for mixing the monomers.

Monomers	0.5 µl each
Vector	0.5 µl
0.5-M Tris-HCl (pH 9.0)	0.5 µl
3% I_2 / EtOH*	0.5 µl

*We use iodine-tincture (Showa-Seiyaku, Japan)

Incubate on 75°C for 3 min and then gradually decrease the temperature to 25°C. Directly transform to DH5a *Escherichia coli* (Takara) and clone on kanamycin plate.

Checking colonies

Conduct colony check PCR.

KOD-FX (Toyobo)	0.2 µl (0.2 U)
2x PCR Buffer	5 µl
2-mM dNTPs	2 µl
10-µM primer	
TALE_monoF	0.25 µl
TALE_monoR4	0.25 µl
H_2O	up to 10 µl

Conduct PCR as follows:

94°C for 2 min

(30 cycles of)

96°C for 10 s

60°C for 15 s

68°C for 1 min

68°C for 2 min

Confirm sizes by electrophoresis. We also recommend checking sequences using either of the primers used in the PCR. Liquid-culture successful clones of each module.

TALEN assembly

Purify the plasmids from the liquid culture. We use Wizard® Plus Minipreps DNA Purification System (Promega) for plasmid purification.

Chose a proper backbone plasmid and mix the following regents.

Hexamer/pentamer plasmids	100 ng each
Backbone plasmid	100 ng
*Bsa*I (NEB)	10 U
Ligation High 2 (Toyobo)	5 μl
H_2O	up to 10 μl

Reaction conditions are as follows:

(10 cycles of)

37°C for 5 min

16°C for 5 min

50°C for 5 min

80°C for 5 min

Transform to DH5a *E. coli* and clone on ampicillin plate.

Sequence Check

The sequence of a constructed TALEN can be checked with the three sequencing primers TALE-Seq-F1, TALE-Seq-R3, and TALE-Seq-R4 for the 1−6th, 7−12th and 13−18th repetitive units, respectively.

Reference

Sanjana NE, Cong L, Zhou Y, Cunniff MM, Feng G, Zhang F (2012). A transcription activator-like effector toolbox for genome engineering. *Nature protocols* **7:** 171-192.
